# A Novel Epidemic Model for Wireless Rechargeable Sensor Network Security

**DOI:** 10.3390/s21010123

**Published:** 2020-12-27

**Authors:** Guiyun Liu, Baihao Peng, Xiaojing Zhong

**Affiliations:** 1 School of Mechanical and Electric Engineering, Guangzhou University, Guangzhou 510006, China; liugy@gzhu.edu.cn (G.L.); zhongxj@gzhu.edu.cn (X.Z.); 2 School of Electronics and Communication Engineering, Guangzhou University, Guangzhou 510006, China

**Keywords:** wireless rechargeable sensor network, cyber security, stability analysis, optimal control

## Abstract

With the development of wireless rechargeable sensor networks (WRSNs ), security issues of WRSNs have attracted more attention from scholars around the world. In this paper, a novel epidemic model, SILS(**S**usceptible, **I**nfected, **L**ow-energy, **S**usceptible), considering the removal, charging and reinfection process of WRSNs is proposed. Subsequently, the local and global stabilities of disease-free and epidemic equilibrium points are analyzed and simulated after obtaining the basic reproductive number $R_{0}$. Detailedly, the simulations further reveal the unique characteristics of SILS when it tends to being stable, and the relationship between the charging rate and $R_{0}$. Furthermore, the attack-defense game between malware and WRSNs is constructed and the optimal strategies of both players are obtained. Consequently, in the case of $R_{0}<1$ and $R_{0}>1$, the validity of the optimal strategies is verified by comparing with the non-optimal control group in the evolution of sensor nodes and accumulated cost.

## 1. Introduction

### 1.1. Research Background

Wireless sensor networks (WSNs) have attracted researchers’ attention worldwide over the last few years. WSNs consist of sensor nodes that have data storing and data transmitting capacities in the form of multi-hop or single-hop. Sensor nodes are randomly deployed in unattended areas in order to monitor the physical environment in and around their vicinity. WSNs have extremely wide applications that range from people’s daily life to various manufacturing industries, and even military facilities, such as health care services, bridge monitoring, intrusion detection, and security surveillance [1]. However, WSNs suffer from various issues related to security [2] and short life cycle [3], due to the vulnerability of the network structure and battery limitations.

Wireless rechargeable sensor networks (WRSNs), as an emerging technology, lie in the breakthrough in wireless power transfer (WPT) technology. It solves the problems of limited energy storage capacity and inconvenient battery replacement, which greatly develop wireless sensor networks (WSNs). So far, WRSNs have carried out a large number of relevant studies and applied research. In recent years, studies on WRSNs mainly focus on charging scheduling and system performance optimizations [4,5,6] However, security issues in WRSNs are seldom concerned by scholars. Malware, as a self-replicating malicious code, once implanted in the networks can cause information leakage and even network paralysis. Specifically, due to the particularity of rechargeability, rechargeable sensor nodes also suffer from the Denial of Charge (DOC) attacks [7], which will cause catastrophic consequence to real-time and pre-waring application fields [8]. Thus, research on WRSNs’ security is urgent and important.

### 1.2. Related Work

The security of data transmission links(DTLs), as an essential part of data transmission, is one of the security issues of WSNs. For example, a cloud using multi-sinks (CPSLP) scheme has been proposed to solve the problem of source location privacy [9] in DTLs. Similarly, in WRSNs, Shafie et al. [10] and Bhushan et al. [11] propose a novel efficient scheme and Energy Efficient Secured Ring Routing (E2SR2) protocols, respectively, in order to enhance the security performance of WRSNs. Besides, as the key hub of DTLs, security on a cluster head draws academic attention [12,13].

Meanwhile, intrusion detection systems (IDSs) have been recognized as the most effective means of detecting malicious attack (Blaster, SDBot, Fork bomb, etc. [14]). Specifically, Cui et al. [15] propose a mobile malware detection systems. Jaint et al. [16] and Thaile et al. [17] apply support a vector machine and nodetrust scheme in order to improve the detection efficiency. Once the malware is detected, then a mitigation mechanism, such as dismissing the affected nodes [18] or adopting diverse variants deployments [19], would be activated.

The application of epidemic dynamics to study the propagation of malware (Worms, Botnets, Rabbit, etc. [14]) in WSNs has received extensive attention and in-depth exploration in the academic. When considering the malware carrier, patch injection mechanism, and time delay, more suitable epidemic models have been proposed: SCIRS [20], SIPS [21], SEIAR [22], etc. Furthermore, Table 1 lists some relevant literature from recent years.

Differential games are also widely used in WSNs as a method for studying optimal dynamic strategies. For example, by using the differential game framework, Al-Tous et al. [29] propose an efficient scheme for power control and data scheduling of energy-harvesting WSNs and Huang et al. [30] develop a virus-resistant weight adaption scheme for mitigating the spread of malware in the large-scale complex networks. Similarly, Table 2 summarizes a few related literatures.

However, as far as we know, the theories of epidemic dynamics and differential game that are applied in WRSNs’ security are rarely studied. Therefore, this paper applies these two theories to study the security of WRSNs to provide a novel perspective of solution.

### 1.3. Contributions

The main goal of this paper is to introduce a novel epidemic model when considering the residual energy of sensor nodes and classify sensor nodes into five various states. Moreover, charging process and removing process are taken into account. Furthermore, the stability theory and differential game theory have both been applied in order to analyze the characteristics of the evolution of sensor nodes in various states. Our contributions are stated, as follows:

- An epidemic model suitable for WRSNs is designed.
- Based on the next-generation matrix method, the basic reproductive number $R_{0}$ of the system is obtained.Subsequently, by applying the Routh Criterion, Lyapunov function, and the other analytical methods, the local and global stabilities of the disease-free equilibrium solution and the epidemic equilibrium solution are proved and simulated. Moreover, the linear relationship between the state variables when the system tends to be stabilized, and the positive relationship between $R_{0}$ and the charging rate is disclosed in the simulation section.
- Applying the Protryagin Maximum Principle, the optimal game strategy between malware and WRSNs is given. Moreover, by comparing the evolution of sensor nodes in various states and overall cost with the control group, the validity of the strategies is verified in the case of $R_{0}<1$ and $R_{0}>1$.

The rest of the paper is organized, as follows: the main characteristics of the model are present in Section 2; theorems of the local and global stability, and the optimal strategies are proved in Section 3; the simulation results are showed in Section 4; and the conclusions are presented in Section 5.

## 2. Modeling

### 2.1. Epidemic Modeling on WRSNs

**Figure 1:**
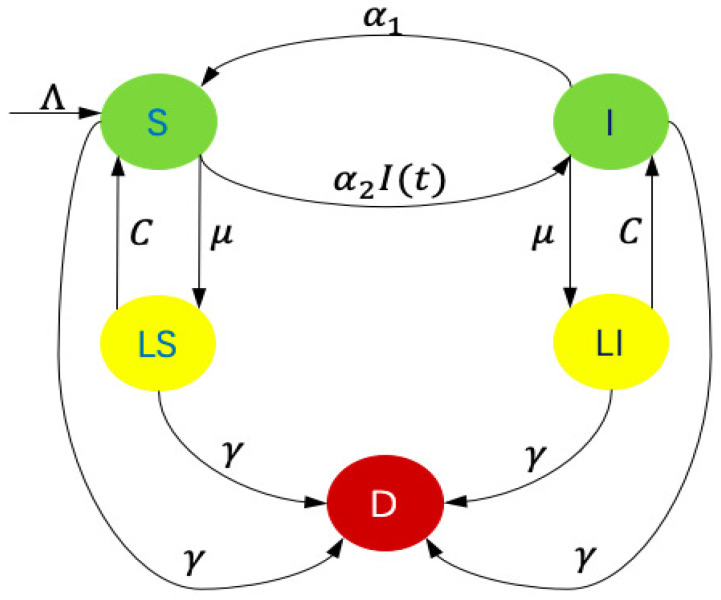
Flow diagram of the **S**usceptible, **I**nfected, **L**ow-energy, **S**usceptible (SILS) Model.

The model presented in this manuscript is global and deterministic such that sensor nodes are classified into five compartments: Susceptible (*S*), Infected (*I*), Susceptible in Low-energy (LS), Infected in Low-energy (LI), and Dysfunctional (*D*), as shown in Figure 1. Sensor nodes in *S* are vulnerable to malware; sensor nodes in *I* are compromised with malware and perform malicious action; sensor nodes in LS and LI state are both in dormant-like state; and, the sensor nodes in *D* state are completely incapacitated, owing to irreparable hardware damage. Specifically, the dormant-like state indicates the sensor nodes are forced to stop some running modules due to the low remaining energy, including data transmission. Thus, sensor nodes in LI state do not have the risk of spreading malware.

Sensor nodes in *S* transform to *I* once the attached malware starts running. New infectious sensor nodes are generated with $\alpha_{2}I\left(t\right)S\left(t\right)$ and $\alpha_{2}$ represents the transmission coefficient. In addition to the new sensor nodes, which are governed by Λ, some of the sensor nodes in *I* are repaired and converted to *S* at rate $\alpha_{1}$. Owing to electricity consumption, sensor nodes in both *S* and *I* drop to low-energy at rate μ. Meanwhile, when considering rechargeable battery equipped in sensor nodes and charging sensor nodes by multiple wireless charging vehicles [41], low-energy sensor nodes rise back to their previous states, since their batteries are full of electricity again. Detailedly, in order to simplify the model, assuming that the charging rate *C* is a constant. The model is simplified by assuming the same mortality μ in each compartment. In the Abbreviation Section, a brief description of the parameters is shown.

As a consequence, the dynamics of the system is governed by (1)–(5):

(1) $$\dot{S(t)}=\Lambda -\left[\alpha_{2}I\left(t\right)+\mu +\gamma \right]S\left(t\right)+\alpha_{1}I\left(t\right)+CLS\left(t\right),$$

(2) $$\dot{I(t)}=\left[-\alpha_{1}-\mu -\gamma +\alpha_{2}S\left(t\right)\right]I\left(t\right)+CLI\left(t\right),$$

(3) $$\dot{LI(t)}=-\left(C+\gamma \right)LI\left(t\right)+\mu I\left(t\right),$$

and

(4) $$\dot{LS(t)}=-\left(C+\gamma \right)LS\left(t\right)+\mu S\left(t\right),$$

(5) $$\dot{D(t)}=\gamma \left[S\left(t\right)+I\left(t\right)+LS\left(t\right)+LI\left(t\right)\right].$$

Moreover, N(t)=S(t)+I(t)+LS(t)+LI(t) and constrained by:

(6) $$\dot{N(t)}=\Lambda -\gamma N\left(t\right).$$

As *t* tends to infinity, considering LS(t)=N(t)−S(t)−I(t)−LI(t), the feasible region obtained is

$$\Omega =\{\left(S,\;I,\;LI\right)\in R^{3}|\;0\leq S,\;I,\;LI\leq \frac{\Lambda }{\gamma }\}.$$

Then, we use the following (7)–(10) to define its boundary:

(7) $$F_{1}=\left\{\left(S,\;I,\;LI\right)\in R^{3}|\;S+I+LI=\frac{\Lambda }{\mu },\;0\leq S,\;I,\;LI\leq \frac{\Lambda }{\gamma }\right\},$$

(8) $$F_{2}=\left\{\left(S,\;I,\;LI\right)\in R^{3}|\;S=0,\;0\leq I,\;LI\leq \frac{\Lambda }{\gamma }\right\},$$

(9) $$F_{3}=\left\{\left(S,\;I,\;LI\right)\in R^{3}|\;I=0,\;0\leq S,\;LI\leq \frac{\Lambda }{\gamma }\right\},$$

and

(10) $$F_{4}=\left\{\left(S,\;I,\;LI\right)\in R^{3}|\;LI=0,\;0\leq S,\;I\leq \frac{\Lambda }{\gamma }\right\}.$$

Considering the following equations:

(11) $${\left(\dot{S}\left(t\right),\;\dot{I}\left(t\right),\;\dot{LI}\left(t\right)\right)}_{F_{1}}\cdot \left(1,\;1,\;1\right)=-\mu S\left(t\right)\leq 0,$$

(12) $${\left(\dot{S}\left(t\right),\;\dot{I}\left(t\right),\;\dot{LI}\left(t\right)\right)}_{F_{2}}\cdot \left(-1,\;0,\;0\right)=-\Lambda -C\left[N-I\left(t\right)-LI\left(t\right)\right]\leq 0,$$

(13) $${\left(\dot{S}\left(t\right),\;\dot{I}\left(t\right),\;\dot{LI}\left(t\right)\right)}_{F_{3}}\cdot \left(0,\;-1,\;0\right)=-CLI\left(t\right)\leq 0,$$

and

(14) $${\left(\dot{S}\left(t\right),\;\dot{I}\left(t\right),\;\dot{LI}\left(t\right)\right)}_{F_{4}}\cdot \left(0,\;0,\;-1\right)=-\mu I\left(t\right)\leq 0.$$

Consequently, Ω is compact and invariant [42], and the solutions of Ω exist and are unique [43].

### 2.2. Computation of the Steady States and the Basic Reproductive Number $R_{0}$

Considering the limit system, we obtain:

(15) $$\dot{S(t)}=\Lambda -\left[\alpha_{2}I\left(t\right)+\mu +\gamma \right]S\left(t\right)+\alpha_{1}I\left(t\right)+C\left[N-S\left(t\right)-I\left(t\right)-LI\left(t\right)\right],$$

(16) $$\dot{I(t)}=\left[-\alpha_{1}-\mu -\gamma +\alpha_{2}S\left(t\right)\right]I\left(t\right)+CLI\left(t\right),$$

and

(17) $$\dot{LI(t)}=-\left(C+\gamma \right)LI\left(t\right)+\mu I\left(t\right),$$

where $N=\lim_{t\to \infty }N\left(t\right)=\frac{\Lambda }{\gamma }$.

The solutions of the above system are the steady states of (1)–(5). This system has two solutions:

1. The disease-free steady states $E_{0}=\left(S_{0},0,0\right)$, where
  (18) $$S_{0}=\frac{C\Lambda +\gamma \Lambda }{C\gamma +\gamma \mu +\gamma^{2}}.$$
2. The endemic steady state $E^{*}=\left(S^{*},I^{*},LI^{*}\right)$, where
  (19) $$S^{*}=\frac{\left(\alpha_{1}+\mu +\gamma \right)\left(C+\gamma \right)-C\mu }{\alpha_{2}\left(C+\gamma \right)},$$
  (20) $$I^{*}=\frac{\left[\left(\mu +\gamma \right)\left(C+\gamma \right)-C\mu \right]\left[\left(\alpha_{1}+\mu +\gamma \right)\left(C+\gamma \right)-C\mu \right]-\Lambda \alpha_{2}{\left(C+\gamma \right)}^{2}}{-\alpha_{2}\left(C+\gamma \right)\left[\left(\alpha_{1}+\mu +\gamma \right)\left(C+\gamma \right)-C\mu \right]+\alpha_{1}\alpha_{2}{\left(C+\gamma \right)}^{2}},$$
  and
  (21) $$LI^{*}=\frac{\mu \left[\left(\mu +\gamma \right)\left(C+\gamma \right)-C\mu \right]\left[\left(\alpha_{1}+\mu +\gamma \right)\left(C+\gamma \right)-C\mu \right]-\Lambda \mu \alpha_{2}{\left(C+\gamma \right)}^{2}}{-\alpha_{2}{\left(C+\gamma \right)}^{2}\left[\left(\alpha_{1}+\mu +\gamma \right)\left(C+\gamma \right)-C\mu \right]+\alpha_{1}\alpha_{2}{\left(C+\gamma \right)}^{3}}.$$

Furthermore, the basic reproductive number $R_{0}$ can be obtained by the next generation matrix method [44].

Set

(22) $$F=\left(\begin{array}{cc} \alpha_{2}S\left(t\right) & 0\\ 0 & 0 \end{array}\right)$$

and

(23) $$V=\left(\begin{array}{cc} \alpha_{1}+\mu +\gamma & -C\\ -\mu & C+\gamma \end{array}\right).$$

Thus

(24) $$\begin{array}{cc} R_{0}= & F\cdot V^{-1}=\frac{\alpha_{2}S_{0}\left(C+\gamma \right)}{\left(\alpha_{1}+\mu +\gamma \right)\left(C+\gamma \right)-C\mu }\\ = & \frac{\alpha_{2}\Lambda {\left(C+\gamma \right)}^{2}}{\left[\left(C+\gamma \right)\left(\mu +\gamma \right)-C\mu \right]\left[\left(\alpha_{1}+\mu +\gamma \right)\left(C+\gamma \right)-C\mu \right]}. \end{array}$$

## 3. Dynamic Analysis

In this section, the local and global stabilities of both the disease-free point $E_{0}$ and the epidemic equilibrium point $E^{*}$ were fully proved. Moreover, an attack-defense game was built to analyze the confrontation between malware and WRSNs.

### 3.1. Local Stability Analysis

**Theorem** **1.** *The disease-free equilibrium point, $E_{0}$, is locally asymptotically stable if $R_{0}<1$.*

**Proof.**  The eigenvalues of the following matrix

(25) $$F-V=\left(\begin{array}{cc} \alpha_{2}S_{0}-\left(\alpha_{1}+\mu +\gamma \right) & C\\ \mu & -(C+\gamma ) \end{array}\right)$$

are

(26) $$\lambda_{1}=0.5\left(-b+\sqrt{b^{2}-4c}\right),$$

and

(27) $$\lambda_{2}=0.5\left(-b-\sqrt{b^{2}-4c}\right),$$

where $b=\left(C+\gamma \right)-[\alpha_{2}S_{0}-\left(\alpha_{1}+\mu +\gamma \right)]$ and $c=[\left(C+\gamma \right)\left(\alpha_{2}S_{0}-\alpha_{1}-\mu -\gamma \right)+C\mu ]$. The real parts of the two eigenvalues are both negative if $R_{0}<1$. Besides,

(28) $$\frac{\partial \left[-\mu S(t)-\gamma S(t)+C(N-S)+\Lambda \right]}{\partial S}=-\mu -\gamma -C<0.$$

Thus $E_{0}$ is locally asymptotically stable [45]. On the contrary, $E_{0}$ is unstable if $R_{0}>1$. □

**Theorem** **2.** *The epidemic equilibrium point, $E^{*}$, is locally asymptotically stable if $R_{0}>1$.*

**Proof.**  First of all, the truth $E^{*}$ exists if and only if $R_{0}>1$ is simple to prove.

Subsequently, the characteristic polynomial of the Jacobian matrix of the state functions (1)–(4) in $E^{*}$, when $R_{0}>1$ is

(29) $$P\left(\lambda \right)=P_{1}\lambda^{3}+P_{2}\lambda^{2}+P_{3}\lambda^{1}+P_{4}\lambda^{0},$$

where

(30) $$P_{1}=1>0,$$

(31) $$P_{2}=\mu +2\gamma +2C+\frac{\alpha_{2}^{2}\left(C+\gamma \right)\left(C\gamma +\gamma \mu +\gamma^{2}\right)\left(R_{0}-1\right)}{\left(C\gamma +\gamma^{2}+\mu \gamma \right)\left(C\alpha_{1}+\alpha_{1}\gamma +\mu \gamma +C\gamma +\gamma^{2}\right)}+\frac{C\mu }{C+\gamma }>0,$$

(32) $$P_{3}=\gamma \left(\mu +\gamma \right)+\frac{\alpha_{2}^{2}{\left(C+\gamma \right)}^{2}\left(C\gamma +\gamma \mu +\gamma^{2}\right)\left(R_{0}-1\right)}{\left(C\gamma +\gamma^{2}+\mu \gamma \right)\left(C\alpha_{1}+\alpha_{1}\gamma +\mu \gamma +C\gamma +\gamma^{2}\right)}+\frac{C\mu }{C\left(C+2\gamma +\mu \right)}>0,$$

and

(33) $$P_{4}=\frac{\mu C\alpha_{2}^{2}\left(C+\gamma \right)\left(C\gamma +\gamma \mu +\gamma^{2}\right)\left(R_{0}-1\right)}{\left(C\gamma +\gamma^{2}+\mu \gamma \right)\left(C\alpha_{1}+\alpha_{1}\gamma +\mu \gamma +C\gamma +\gamma^{2}\right)}>0.$$

Moreover, a simple calculus shows $P_{2}P_{3}-P_{1}P_{4}>0$. Thus, if $R_{0}>1$, applying the Routh criterion [46], the local asymptotically stability of $E^{*}$ follows. □

### 3.2. Global Stability Analysis

**Theorem** **3.** *The disease-free equilibrium point, $E_{0}$, is globally asymptotically stable if $R_{0}<1$.*

**Proof.**  In this proof, the method of Lyapunov function is considered. In general, in the Lyapunov stability analysis [47], the Lyapunov function needs to be positive definite, except for the stable point, and its first derivative needs to be negative definite.

When considering the Lyapunov function V=(C+γ)I(t)+CLI(t), we obtain:

(34) $$\begin{array}{cc} \dot{V(t)} & =\left(C+\gamma \right)\dot{I(t)}+C\dot{LI(t)}\\ & =\left(C+\gamma \right)\left\{I\left(t\right)\left[\alpha_{2}S\left(t\right)-\left(\alpha_{1}+\gamma +\mu \right)\right]+CLI\left(t\right)\right\}-C\left(C+\gamma \right)LI\left(t\right)+C\mu I\left(t\right)\\ & \leq \left(C+\gamma \right)I\left(t\right)\left[\alpha_{2}S_{0}-\left(\alpha_{1}+\gamma +\mu \right)\right]+C\mu I\left(t\right)\\ & =I\left(t\right)[\left(C+\gamma \right)\alpha_{2}S_{0}-\left(C+\gamma \right)\left(\alpha_{1}+\gamma +\mu \right)+C\mu ]\\ & =I\left(t\right)\left(R_{0}-1\right) \end{array}$$

In addition, $\frac{dV}{dt}=0$ if and only if $R_{0}=1$ and I(t)=0. Moreover, (*S*, *I*, LI) tends to $E_{0}$ when *t* tends to infinity, and the maximum invariant set in $\left\{\left(S,I,LI\right)\in \Omega :\frac{dV}{dt}=0\right\}$ is $E_{0}$. Thus, Theorem 3 has been proved, after considering the La-Salle Invariance Principle [48]. □

**Theorem** **4.** *The epidemic equilibrium point, $E^{*}$, is globally asymptotically stable if $R_{0}>1$.*

**Proof.**  First of all, by referencing [49], the system is uniformly persistent. According to [50], there exists an absorbent compact. Besides, according to Theorem 2, $E^{*}$ is the unique equilibrium point if $R_{0}>1$.

The second additive compound matrix of Jacobian matrix is given, as follows:

(35) $$J^{\left[2\right]}=\left(\begin{array}{ccc} \theta_{1}\left(t\right) & C & C\\ \mu & \theta_{2}\left(t\right) & \theta_{3}\left(t\right)\\ 0 & \alpha_{2}I\left(t\right) & \theta_{4}\left(t\right) \end{array}\right).$$

where

(36) $$\theta_{1}\left(t\right)=\alpha_{2}I\left(t\right)-2\mu -2\gamma -C-\alpha_{1}-a+\alpha_{2}S\left(t\right),$$

(37) $$\theta_{2}\left(t\right)=-\alpha_{2}I\left(t\right)-\mu -2\gamma -2C,$$

(38) $$\theta_{3}\left(t\right)=-\alpha_{2}S\left(t\right)+\alpha_{1}-C,$$

and

(39) $$\theta_{4}\left(t\right)=-\alpha_{1}-\mu -2\gamma -a-C+\alpha_{2}S\left(t\right).$$

Set $P_{f}$ as the directional derivative of the diagonal matrix $P=diag(1,\frac{I}{LI},\frac{I}{LI})$, then:

(40) $$P_{f}P^{-1}=diag\left(0,\frac{\dot{I}}{I}-\frac{\dot{LI}}{LI},\frac{\dot{I}}{I}-\frac{\dot{LI}}{LI}\right).$$

Set the matrix $B=P_{f}P^{-1}+PJ^{\left[2\right]}P^{-1}=$

(41) $$\left(\begin{array}{ccc} \theta_{1}\left(t\right) & C & C\\ \mu & \theta_{2}\left(t\right)+\frac{\dot{I}}{I}-\frac{\dot{LI}}{LI} & \theta_{3}\left(t\right)\\ 0 & \alpha_{2}I\left(t\right) & \theta_{4}\left(t\right)+\frac{\dot{I}}{I}-\frac{\dot{LI}}{LI} \end{array}\right).$$

Set

(42) $$B_{11}=\theta_{1}\left(t\right),$$

(43) $$B_{12}=\left(\begin{array}{cc} C & C \end{array}\right),$$

(44) $$B_{21}=\left(\begin{array}{cc} \mu & 0 \end{array}\right),$$

and

(45) $$B_{22}=\left(\begin{array}{cc} \theta_{2}\left(t\right)+\frac{\dot{I}}{I}-\frac{\dot{LI}}{LI} & \theta_{3}\left(t\right)\\ \alpha_{2}I\left(t\right) & \theta_{4}\left(t\right)+\frac{\dot{I}}{I}-\frac{\dot{LI}}{LI} \end{array}\right).$$

Supposing that (S,I,LI) is a vector in $R^{3}$, then the norm of vector that is defined in $R^{3}$ is

(46) |(S,I,LI)|=max{|S|,|I|+|LI|}.

Note that the Lozinskii measure of *B* is given by the following expression:

(47) $$\mu \left(B\right)\leq max\left\{g_{1},g_{2}\right\}.$$

where $g_{1}=\mu \left(B_{11}\right)+\left|B_{12}\right|$, $g_{2}=\left|B_{21}\right|+\mu \left(B_{22}\right)$

According to [51],

(48) $$g_{1}=-\alpha_{2}I\left(t\right)-2\mu -2\gamma -2C-\alpha_{1}+\alpha_{2}S\left(t\right),$$

(49) $$g_{2}=-3\mu -2\gamma -C-\alpha_{1}+\alpha_{2}S\left(t\right).$$

Supposing that C>μ, then $g_{1}<g_{2}$. Thus, $\mu \left(B\right)\leq g_{2}$.

Subsequently,

(50) $$q=\lim_{t\to \infty }sup{sup}_{\left(S(0),I(0),LI(0))\in int(\Omega \right)}\frac{1}{t}\int_{0}^{t}\mu (B)ds\leq -3\mu -2\gamma -C-\alpha_{1}<0.$$

Consequently, applying the theorem in [52], the statement is proved. □

### 3.3. Optimal Control Strategies

According to differential game theory [53], let us impose a set of hypotheses as follows.

(a) The game in this paper consists of two parties, i.e., malware and WRSN.
(b) Both of the parties have controllable means. Among them, $\nu \left(t\right)=\{A_{SI}\left(t\right),A_{LII}\left(t\right)\}$ represents the strength of spreading malware, i.e., $A_{SI}\left(t\right)$, and the controls from LI to *I*, i.e., $A_{LII}\left(t\right)$. $\mu \left(t\right)=\{D_{IS}\left(t\right),D_{LSS}\left(t\right)\}$ describes the strength of removing malware, i.e., $D_{IS}\left(t\right)$, and the controls from LS and *S*, i.e., $D_{LSS}\left(t\right)$. Thus, (1)–(3) are replaced as:
  (51) $$\dot{S(t)}=\Lambda -\left[\alpha_{2}A_{SI}\left(t\right)I\left(t\right)+\mu +\gamma \right]S\left(t\right)+\alpha_{1}D_{IS}\left(t\right)I\left(t\right)+CD_{LSS}\left(t\right)LS\left(t\right),$$
  (52) $$\dot{I(t)}=\left[-\alpha_{1}D_{IS}\left(t\right)-\mu -\gamma +\alpha_{2}A_{SI}\left(t\right)S\left(t\right)\right]I\left(t\right)+CA_{LII}\left(t\right)LI\left(t\right),$$
  (53) $$\dot{LS(t)}=-\left(CD_{LSS}\left(t\right)+\gamma \right)LS\left(t\right)+\mu S\left(t\right),$$
  and
  (54) $$\dot{LI(t)}=-\left(CA_{LII}\left(t\right)+\gamma \right)LI\left(t\right)+\mu I\left(t\right).$$
(c) Define X(t)={S(t),I(t),LS(t),LI(t),D(t)} as a set of state variables.
(d) The attacker (i.e., malware) aims at maximizing J(·) and the defender (i.e., WRSNs) aims at minimizing J(·), and
  (55) $$J\left(t,X\left(t\right),\mu \left(t\right),\nu \left(t\right)\right)=\int_{t_{0}}^{t_{f}}[C_{I}I\left(t\right)]dt+\sum_{i\in X(t)}C_{i(t_{f})}i\left(t_{f}\right),$$
  where $C_{I}$ indicates the cost incurred by *I* nodes at time *t*, $C_{i(t_{f})}$ indicates the terminal cost of corresponding state, and $i(t_{f})$ indicates the number of corresponding state at the terminal moment.

**Theorem** **5.** *There is an optimal control set $\left(\mu^{*}\left(t\right),\nu^{*}\left(t\right)\right)=(\left\{D_{IS}^{*}\left(t\right),D_{LSS}^{*}\left(t\right)\right\},\{A_{SI}^{*}\left(t\right),$$A_{LII}^{*}\left(t\right)\})$ such that*

(56) $$J\left(t,X\left(t\right),\mu^{*}\left(t\right),\nu^{*}\left(t\right)\right)=max_{\nu }min_{\mu }J\left(t,X\left(t\right),\mu \left(t\right),\nu \left(t\right)\right)=min_{\mu }max_{\nu }J\left(t,X\left(t\right),\mu \left(t\right),\nu \left(t\right)\right)$$

*and the values of $A_{SI}^{*}$ and $D_{IS}^{*}$ follow (57)–(60)*

(57) $$A_{SI}^{*}\left(t\right)=\left\{\begin{array}{ccc} maxA_{SI}\left(t\right) & \; & \Delta_{1}>0\\ minA_{SI}\left(t\right) & \; & \Delta_{1}<0 \end{array}\right.$$

(58) $$A_{LII}^{*}\left(t\right)=\left\{\begin{array}{ccc} maxA_{LII}\left(t\right) & \; & \Delta_{2}<0\\ minA_{LII}\left(t\right) & \; & \Delta_{2}>0 \end{array}\right.$$

(59) $$D_{IS}^{*}\left(t\right)=\left\{\begin{array}{ccc} maxD_{IS}\left(t\right) & \; & \Delta_{3}<0\\ minD_{IS}\left(t\right) & \; & \Delta_{3}>0 \end{array}\right.$$

(60) $$D_{LSS}^{*}\left(t\right)=\left\{\begin{array}{ccc} maxD_{LSS}\left(t\right) & \; & \Delta_{4}<0\\ minD_{LSS}\left(t\right) & \; & \Delta_{4}>0 \end{array}\right.$$

*where $\Delta_{1}=\left[\lambda_{I}\left(t\right)-\lambda_{S}\left(t\right)\right]\alpha_{2}S\left(t\right)I\left(t\right)$, $\Delta_{2}=\left[\lambda_{I}\left(t\right)-\lambda_{LI}\left(t\right)\right]CLI\left(t\right)$, $\Delta_{3}=\left[\lambda_{S}\left(t\right)-\lambda_{I}\left(t\right)\right]$$\alpha_{1}I\left(t\right)$ and $\Delta_{4}=\left[\lambda_{S}\left(t\right)-\lambda_{LS}\left(t\right)\right]CLS\left(t\right)$.*

**Proof.**  The saddle point in the game exists and it is unique [53]. Subsequently, referencing [54], the game has a value *V*, such that

(61) $$V=max_{\nu }min_{\mu }J\left(t,X\left(t\right),\mu \left(t\right),\nu \left(t\right)\right)=min_{\mu }max_{\nu }J\left(t,X\left(t\right),\mu \left(t\right),\nu \left(t\right)\right)=J\left(t,X\left(t\right),\mu^{*}\left(t\right),\nu^{*}\left(t\right)\right).$$

In view of (1)–(5) and (55), the Hamiltonian function is constructed as:

(62) $$\begin{array}{cc} H(t,X(t),\lambda (t),\mu (t),\nu (t))= & \lambda_{S}\left(t\right)\dot{S(t)}+\lambda_{I}\left(t\right)\dot{I(t)}+\lambda_{LS}\left(t\right)\dot{LS(t)}+\lambda_{LI}\left(t\right)\dot{LI(t)}\\ & +\lambda_{D}\left(t\right)\dot{D(t)}+C_{I}I\left(t\right) \end{array}$$

where $\lambda \left(t\right)=\{\lambda_{S}\left(t\right),\lambda_{I}\left(t\right),\lambda_{LS}\left(t\right),\lambda_{LI}\left(t\right),\lambda_{D}\left(t\right)\}$ is a set of co-state variables.

By applying the Pontryagin Maximum Principle [55], the constraints of the co-state variables are formulated, as follows.

(63) $$\dot{\lambda_{S}\left(t\right)}=\left[\lambda_{S}\left(t\right)-\lambda_{I}\left(t\right)\right]\alpha_{2}A_{SI}\left(t\right)I\left(t\right)+\left[\lambda_{S}\left(t\right)-\lambda_{LS}\left(t\right)\right]\mu +\left[\lambda_{S}\left(t\right)-\lambda_{D}\left(t\right)\right]\gamma$$

(64) $$\dot{\lambda_{I}\left(t\right)}=\left[\lambda_{S}\left(t\right)-\lambda_{I}\left(t\right)\right]\alpha_{2}A_{SI}\left(t\right)S\left(t\right)+\left[\lambda_{I}\left(t\right)-\lambda_{S}\left(t\right)\right]\alpha_{1}D_{IS}\left(t\right)+\left[\lambda_{I}\left(t\right)-\lambda_{LI}\left(t\right)\right]\mu -C_{I}$$

(65) $$\dot{\lambda_{LS}\left(t\right)}=\left[\lambda_{LS}\left(t\right)-\lambda_{S}\left(t\right)\right]CD_{LS}\left(t\right)+\left[\lambda_{LS}\left(t\right)-\lambda_{D}\left(t\right)\right]\gamma$$

(66) $$\dot{\lambda_{LI}\left(t\right)}=\left[\lambda_{LI}\left(t\right)-\lambda_{I}\left(t\right)\right]CA_{LI}\left(t\right)+\left[\lambda_{LI}\left(t\right)-\lambda_{D}\left(t\right)\right]\gamma$$

(67) $$\dot{\lambda_{D}\left(t\right)}=0$$

Besides, the terminal constraints of the co-state variables are formulated as:

(68) $$\lambda_{i}\left(t_{f}\right)=C_{i(t_{f})}$$

where i∈X(t).

Consequently, the optimal strategies are obtained by

(69) $$H\left(t,X^{*}\left(t\right),\lambda \left(t\right),\mu^{*}\left(t\right),\nu \left(t\right)\right)\leq H\left(t,X^{*}\left(t\right),\lambda \left(t\right),\mu^{*}\left(t\right),\nu^{*}\left(t\right)\right)\leq H\left(t,X^{*}\left(t\right),\lambda \left(t\right),\mu \left(t\right),\nu^{*}\left(t\right)\right).$$

□

As a consequence, in the optimal case, when $\left[\lambda_{I}\left(t\right)-\lambda_{S}\left(t\right)\right]\alpha_{2}S\left(t\right)I\left(t\right)>0$, the malware exerts maximum effort to infect vulnerable sensor nodes; otherwise, it does not propagate. When $[\lambda_{I}\left(t\right)-\lambda_{LI}\left(t\right)]CLI\left(t\right)<0$, the malware exerts the minimum effort to influence the charging process to LI nodes; otherwise, the LI nodes accept the charging requests. Moreover, when $\left[\lambda_{S}\left(t\right)-\lambda_{I}\left(t\right)\right]\alpha_{1}I\left(t\right)<0$, WRSNs exert the maximum effort to clear the malware; otherwise, the networks does nothing in removing malware. When $[\lambda_{S}\left(t\right)-\lambda_{LS}\left(t\right)]CLS\left(t\right)<0$, WRSNs exert the maximum effort to charge the LS nodes; otherwise, LS nodes do not charge.

## 4. Simulation

Theorem 1 to Theorem 5 have been further verified here. In Section 4.1, the stable solutions of the system (1)–(5) are obtained and proved, while the impact of charging is analyzed by observing the variation of *I* nodes. In Section 4.2, the optimal solutions are displayed by comparing with the groups without optimal controls. All of the simulations are based on MacOS Catalina (Intel Core i5, 8 GB, 1.8 GHz) and MATLAB 2017b.

### 4.1. Stability Analysis

This part aims to test Theorem 1 to Theorem 4. Firstly, eight two-dimensional feasible regions are constructed through the combinations of various state variables to analyze their relationships. Subsequently, suppose that Λ=0.1, γ=0.005, μ=0.05, $\alpha_{2}=0.001$, $\alpha_{1}=0.01$, and C=0.05. Therefore, whether $R_{0}<1$ or $R_{0}>1$, the total number of sensor nodes is constant at 20 (i.e., S(t)+I(t)+LS(t)+LI(t)≤20), including the initial value (i.e., S(0)+I(0)+LI(0)+LS(0)=20).

#### 4.1.1. Disease-Free Equilibrium Stability

**Figure 2:**
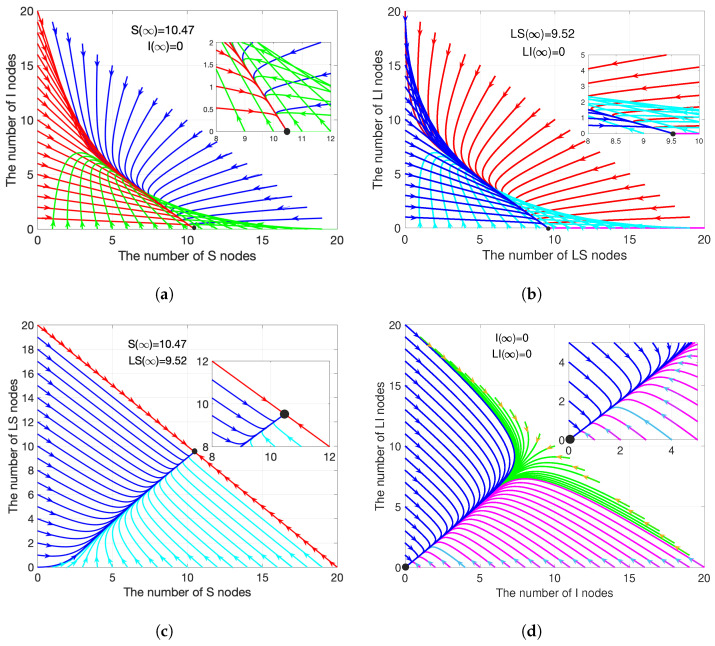
Evolution of sensor nodes when $R_{0}<1$. Different colors indicate that the curves start at different boundaries. So do Figure 3, Figure 4 and Figure 5.

Here, four different state combinations (i.e., (S(t),I(t)),(LS(t),LI(t)),(S(t),LS(t)), and (I(t),LI(t))) are given in order to testify the disease-free equilibrium point, as shown in Figure 2. At this moment, $R_{0}=0.5360<1$. Theoretically, S(∞)=10.47, I(∞)=LI(∞)=0, and LS(∞)=9.52, according to (18). Practically, the simulation results that are shown in Figure 2 conform to Theorems 1 and 3.

The evolution in (S(t),I(t)) and (LS(t),LI(t)) are similar and so do (S(t),LS(t)) and (I(t),LI(t)), as depicted in Figure 2. In Figure 2a,b, the curve starts from the boundary and finally converges to (10.47, 0)((9.52, 0)). Specifically, as illustrated in Figure 2a,b, the curve in the feasible region is attracted by I=−1.0028S+10.5(LI=−0.9979LS+9.5). When malware only exists on LI nodes, a peak in the number of *I* nodes appears after a period of time. The peak decreases as the initial number of *S* nodes increases, and it eventually stays around at 3. At the same time, the number of *I* nodes always shows a downward trend, and it is cleared when t→∞. In Figure 2c,d, the curve is attracted by LS=1.1240S−2.2479(LI=I), and finally converges to (10.47, 9.52)(0, 0). From the beginning, an increase in the number of *S*(LS) or *I*(LI) nodes must lead to a decrease in the number of LS(*S*) or LI(*I*) nodes, as shown in Figure 2c,d. Subsequently, the number of *S* and LS nodes increase simultaneously, while the number of *I* and LI decrease simultaneously. Consequently, in WRSNs, only *S* and LS nodes exist.

**Figure 3:**
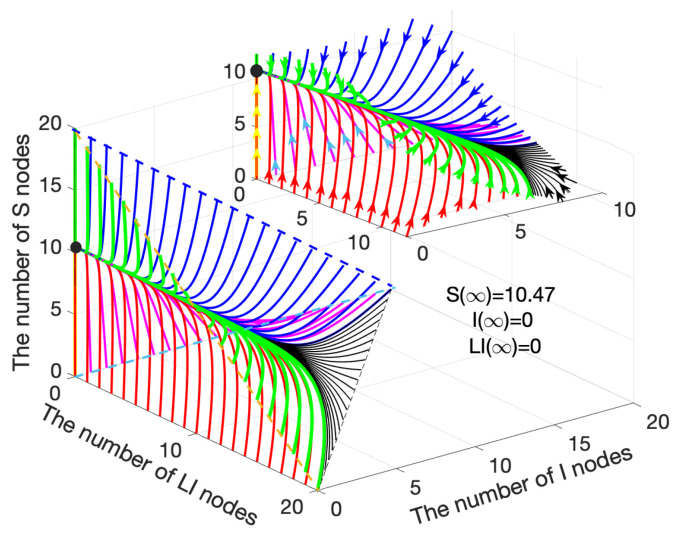
Variation of S, I and LI nodes when $R_{0}<1$.

Figure 3 illustrates a three-dimensional diagram of the system(15)–(17), where the feasible region is in the triangular pyramid area. Note that Figure 2a,d are two feasible region planes in Figure 3. Similarly, the curve gathers together, and then finally converges to (10.47, 0, 0). In summary, a smooth transitional period exists before the system reaches equilibrium. During the period, malware is eliminated from the network gradually and the numbers of *S* and LS nodes maintain a steady growth. In the end, the malware is totally cleared in the WRSNs, and the numbers of *S* and LS nodes remain unchanged under constant charging power.

#### 4.1.2. Epidemic Equilibrium Stability

**Figure 4:**
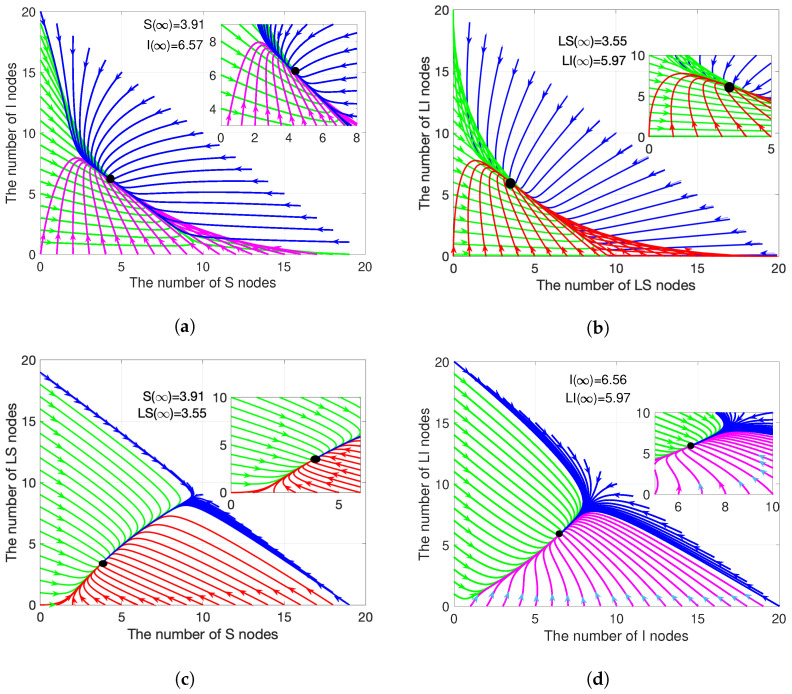
Evolution of sensor nodes when $R_{0}>1$.

**Figure 5:**
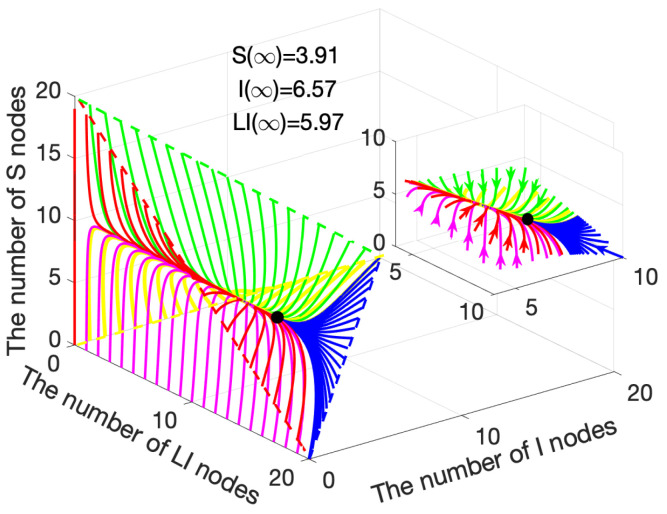
Variation of S, I and LI nodes when $R_{0}>1$.

Similarly, the same four combinations are applied here, as depicted in Figure 4. Suppose that the values of coefficients, except for $\alpha_{2}=0.005$, remain constant, as stated in Section 4.1.1. At this time, $R_{0}=2.6799>1$. Substitute the coefficients into (19)–(21), we obtained $S^{*}=3.91$, $I^{*}=6.57$ and $LI^{*}=5.97$. Moreover, the results that are presented in Figure 5 confirm Theorem 2 and Theorem 4.

When compared with Figure 2, when $R_{0}>1$, the equilibrium points appear inside the region, but not the boundary. In terms of evolution trends, Figure 4a,c and Figure 4b,d are close. It is worth noting that the prerequisite for the existence of the epidemic equilibrium point is that at least one *I* node exists. When compared with the case $R_{0}<1$, at this time, the number of *I*(LI) nodes is no longer reduced. The curve that is shown in Figure 4a,b eventually converges to (3.91, 6.57) (3.55, 5.97). In particular, when the initial number of *I*(LI) nodes is less than 6.57(5.97), the number of *I*(LI) nodes will increase significantly after a gentle decline, as shown in Figure 4a,b. The curve that is presented in Figure 4a,b is attracted by S=−1.1330LS+11(LS=−1.1352LI+10), and it finally converges to the equilibrium point along it.

When compared with the case $R_{0}<1$, the evolution curves of (S(t),LS(t))((I(t),LI(t))) are similar at first. However, when the curves gather, they do not just show a single evolution trend, but the increase and decrease appear at the same time, as shown in Figure 4c,d. In Figure 4c,d, the curve begins from the boundary and it converges to (3.91, 3.55) (6.56, 5.97), when t→∞.

Figure 5 illustrates the limit system (15)–(17) in three-dimensional way. Similarly, Figure 4a,d are the planes into the triangular pyramid region. Similar to the case $R_{0}<1$, the evolution of *S*, *I*, and LI nodes has a smooth transitional period before reaching the equilibrium point. During the transitional period, when the number of S>3.91, the trend drops to (3.91, 6.57, 5.97); when the number of S<3.91, the trend rises to (3.91, 6.57, 5.97). In summary, in the case $R_{0}>1$, if malware propagates on a large scale in WRSNs, after a period of time, the numbers of *I* and LI nodes will decrease along a specific trajectory. Meanwhile, the numbers of *S* and LS nodes increase along a specific trajectory, and finally converge to (3.91, 6.57, 5.97). Conversely, when few malware exists in WRSNs, over a period of evolution, the numbers of *S* and LS nodes decrease along the specific trajectory, and the numbers of *I* and LI increase along the specific trajectory, and finally converge to (3.91, 6.57, 5.97).

#### 4.1.3. Influence of the Charging Rate *C*

**Figure 6:**
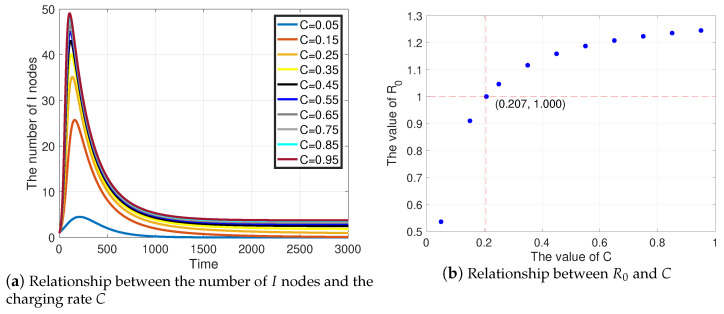
The influence of *C*.

The influence of charging rate is discussed detailedly by comparing the quantity of *I* nodes with various *C*, as shown in Figure 6. Based on (55), *C* directly affects $R_{0}$, thereby affecting the prevalence of malware. As *C* increases, the peak of the quantity of *I* nodes keeps growing and gradually saturates, as shown in Figure 6a. Under ten sets of *C*, only C=0.05 and C=0.15 clear the malware. After simple calculation, we find, as *C* increases, that $R_{0}$ grows likewise, as illustrated in Figure 6b. Specifically, $R_{0}=1.000$, when C=0.207, which indicates, when t→∞, that the malware is eliminated if C<0.207 and prevalent if C>0.207.

Such results reveal that, in WRSNs, in the confrontation with malware, the higher charging power, the higher peak of malware propagation. This inspires us to properly control the charging rate in order to prevent the prevalence of malware.

### 4.2. Optimal Control

In this section, we further classify Theorem 5 into three aspects: evolution of state variables, overall cost and variation of control variables. The coefficients remain constant as set in Section 4.1. Moreover, set $C_{I}=0.05$, S(0)=19, I(0)=1, LS(0)=0, LI(0)=0, $A_{SI}\left(0\right)=1$, $A_{LII}\left(t\right)=0$, $D_{IS}\left(0\right)=1$, and $D_{LSS}\left(0\right)=0$. In particular, this section analyzes the two cases of $R_{0}$ (i.e., $R_{0}<1$ and $R_{0}>1$). Furthermore, the optimality of the strategies (57)–(60) is highlighted by comparison with the non-optimal control groups.

#### Evolution of State Variables

**Figure 7:**
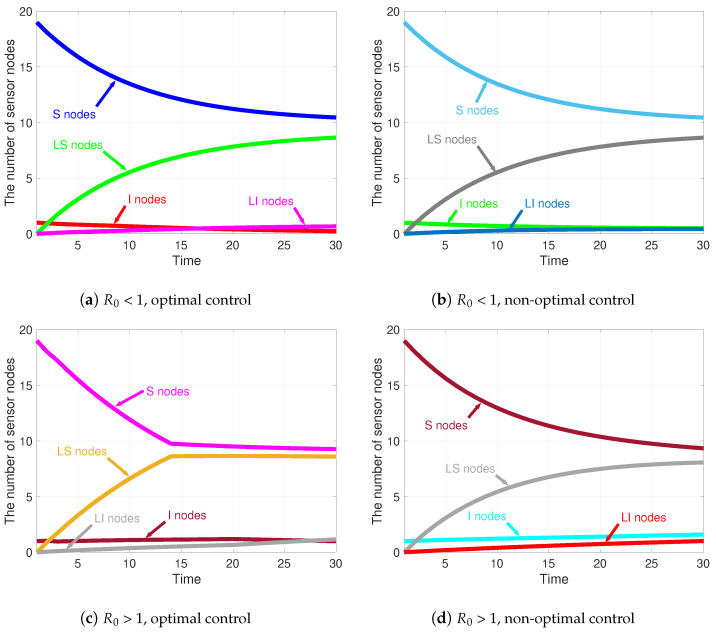
Evolution of sensor nodes under four different cases.

In this part, the evolution of state variables under four cases is discussed, as shown in Figure 7.

Case 1: $R_{0}<1$ with optimal control, as shown in Figure 7a;
Case 2: $R_{0}<1$ without optimal control, as shown in Figure 7b;
Case 3: $R_{0}>1$ with optimal control, as shown in Figure 7c; and,
Case 4: $R_{0}>1$ without optimal control, as shown in Figure 7d.

The evolution trends of *S* and LS nodes are very close in the four cases. Specifically, when t=30, in Case 1, S=10.45 and LS=8.6469; in Case 2, S=10.4412 and LS=8.6379; in Case 3, S=9.2643 and LS=8.5908; and, in Case 4, S=9.3334 and LS=8.0666. When $R_{0}<1$ (i.e., Case 1 and Case 2), the number of *I* nodes always keeps decreasing. However, when $R_{0}>1$ (i.e., Case 3 and Case 4), the number of LI nodes shows increasing signs. As for LI nodes, the number keeps rising in all four cases. Detailedly, when t=30, in Case 1, I=0.2328 and LI=0.6702; in Case 2, I=0.4883 and LI=0.4326; in Case 3, I=0.9919 and LI=1.1530; and, in Case 4, I=1.5960 and LI=1.0039.

The results indicate, in the consecutive attack-defense confrontation game, that the number of *I* nodes decays faster than that of the control groups. Therefore, the **S**usceptible, **I**nfected, **L**ow-energy, **S**usceptible (SILS) model under dynamic optimal controls is more conducive to the clearance of malware in WRSNs.

### 4.3. Overall Cost and Optimal Controls

In this part, the four cases stated above are explained further in two aspects, including accumulative costs and variation on control variables.

**Figure 8:**
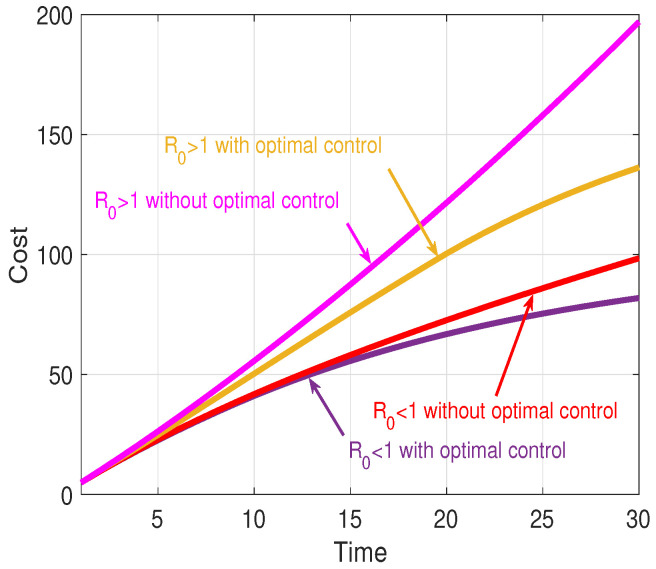
Overall cost.

Figure 8 illustrates the cost under the four cases. From Figure 8, it can be seen that the cost with optimal control is lower than that of the control group all of the time, which indicates the validity. Specifically, in Case 1, the cost finally reaches 81.9044; in Case 2, the cost finally reaches 98.4576; in Case 3, the cost finally reaches 162.8256; and, in Case 4, the cost finally reaches 196.9237. The cost depends on the number of *I* nodes based on (55). Thus, the cumulative cost is another embodiment of variation on the number of *I* nodes. It can be seen, even under optimal control, that the number of *I* nodes when $R_{0}>1$ is still greater than that when $R_{0}<1$.

**Figure 9:**
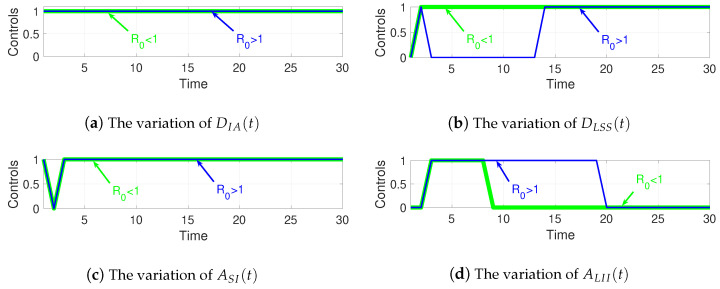
Variation of control variables.

Figure 9 depicts the variations of the control variables when $R_{0}<1$ and $R_{0}>1$ obtained from (57)–(60). Among them, Figure 9a shows the variation of $D_{IA}\left(t\right)$, Figure 9b shows the variation of $D_{LSS}\left(t\right)$, Figure 9c shows the variation of $A_{SI}\left(t\right)$, and Figure 9d shows the variation of $A_{LII}\left(t\right)$.

For WRSNs, its purpose is to minimize the cost. Therefore, whether $R_{0}<1$ or $R_{0}>1$, WRSNs are sparing no effort to remove malware, as depicted in Figure 9a. However, the charging process is not always performed. After charging started, in the case of $R_{0}>1$, owing to the excessive growth of malware, the charging of LS nodes stops immediately, as shown in Figure 9b.

Regarding malware, its aim is to maximize the cost. Thus, in both cases, malware is being copied and propagated almost all of the time, as depicted in Figure 9c. Similarly, LI nodes do not always receive charging requests. If the sharp decline in the number of *I* nodes appears (i.e., t=3 when $R_{0}<1$ and t=19 when $R_{0}>1$), charging LI nodes will cause more malware to be removed, which is unconducive to the spread of the malware.

## 5. Conclusions

A novel model, namely SILS, has been proposed when considering the remaining energy of WRSNs in this paper. In SILS, sensor nodes are divided into five states: susceptible, infected, susceptible in low-energy, infected in low-energy, and dysfunctional. After theoretically proposing local and global stability, we further verify them by simulations. The simulation results indicate some characteristics of the SILS model: before reaching the equilibrium point ($E_{0}$ or $E^{*}$), there exists a phenomenon that the number of *S*(*I*) and LS(LI) nodes increases or decreases linearly simultaneously. Besides, the positive relationship between charging rate *C* and basic reproductive number $R_{0}$ is revealed, which enlightens us to adjust the charging rate *C* reasonably. Meanwhile, the threshold of *C* is obtained, which is, if *C* is higher than the threshold, malware will be prevalent, and below the threshold, malware will be eliminated. In addition to analyzing the stability, by constructing a game model, we further analyze the attack-defense methods of malware and WRSNs, and derive the optimal strategies for both players. At the same time, by comparing the cases $R_{0}>1$ and $R_{0}<1$, the simulation results show that the optimal controls can effectively inhibit the growth of *I* nodes and reduce the overall costs.

This paper discusses a static, homogeneous network. However, with the continuous development of the Internet of Things industry and the integration of various terminal devices, heterogeneous network technology has become mainstream and it is one of our future research areas. At the same time, the convenience that is brought by the mobile technology has became increasingly prominent, such as mobile base stations and mobile chargers. However, the potential safety hazards that are caused by the mobile devices cannot be ignored. Moreover, with the investigation of the actual situation and the deepening of the mathematical model, various random model problems will be raised, which are also the trends in our future research.

## Figures and Tables

**Table 1:** Researches on stability of epidemic model in Wireless sensor networks (WSNs).

Authors	Model	Characteristics	Stability
J.D. HernándezGuillén et al. [20]	SCIRS	Considering the carrier state, population dynamics, and vaccination and reinfection processes	Local and global stability in malware-free and epidemic points
S.G. Shen et al. [23]	VCQPS	Considering both the heterogeneity and mobility of heterogeneous and mobile sensor nodes	Local and global stability in malware-free point
Linhe Zhu et al. [24]	SBD	Considering the nonlinear incidence rate and time delay in complex networks	Local and global stability in rumor-free point
P.K. Srivastava et al. [25]	SEIAR	Considering the anti-malware process	Local and global stability in worm-free point
S. Hosseini et al. [26]	SEIRS-QV	Considering the impacts of user awareness, network delay and diverse configuration of nodes	Local and global stability in malware-free point
D.W. Huang et al. [21]	SIPS	Considering the patch injection mechanism	Local and global stability in epidemic point
L.H. Zhu et al. [22]	I2S2R	Considering the effect of time delay both in homogeneous networks and heterogeneous networks	Local and global stability in malware-free and epidemic points
R.P. Ojha et al. [27]	SEIQRV	Considering both quarantine and vaccination techniques	Local and global stability in worm-free point
S.R. Biswal et al. [28]	SEIRD	Considering the early detection and removal process	Local and global stability in worm-free point

**Table 2:** Researches on differential game applied in WSNs.

Authors	Participants	Goal
H. Al-Tous et al. [29]	An energy-harvesting (EH) multi-hop wireless sensor network (WSN)	Adaptively changing the transmitted data and power, efficiently utilizing the available harvested energy and balancing the buffer of all sensor nodes.
Y.H. Huang et al. [30]	Virus and nodes with various weights	Minimizing the total cost of the whole network
L.T. Zhang, et al. [31]	Device to Device (D2D) offloading enabled mobile network and malware	D2D offloading enabled mobile network aims to maximize the cost
S.G. Shen et al. [32]	WSNs and malware	The systems aims to minimize the cost; the malware aims to maximize the cost (the same cost function)
G.Y. Liu et al. [33]	WRSNs and malware	WRSNs aims to minimize the cost; malware aims to maximize the cost (the same cost)
L. Miao et al. [34]	Intrusion prevention systems(IPS) and the malicious attackers	IPS aims to minimize the cost A; attacker aims to maximize the cost B (two different cost functions )
H.W. Zhang et al. [35]	Attacker and defender	Attacker aims to maximize the cost A; Defender aims to minimize the cost B (two different cost functions)
J.H. Hu et al. [36]	Healthcare-based wireless sensor network (HWSN)	HWSN aims to minimizing the transmission cost
Y. Sun et al. [37]	Edge nodes (ENs)	ENs aims to minimize the resource consumption
S. Eshghi et al. [38]	Mobile WSNs and malware	Mobile WNSs aims to minimize the cost by using optimal patching policies
M.H.R. Khouzani et al. [39]	Mobile WSNs and malware	By obtaining the optimal dissemination of patches, the tradeoff between security risks and bandwidth is minimized
S. Sarkar et al. [40]	Multi-hop wireless networks	By using the optimal routing and scheduling, the throughput of the networks is optimized

## Data Availability

The data presented in this study are available on request from the corresponding author. The data are no publicly available as they involve the subsequent application of patent for invention and the publication of project deliverables.

## Abbreviations

The following abbreviations are used in this manuscript:

Symbol	Description
Λ	Birth rate
γ	Death rate
μ	Depletion rate
$\alpha_{1}$	Removal rate
*C*	Charging rate
$\alpha_{2}$	Transmission rate
